# Liver Resection After Selective Internal Radiation Therapy with Yttrium-90: Safety and Outcomes

**DOI:** 10.1007/s12029-019-00221-0

**Published:** 2019-03-26

**Authors:** Sebastian Mafeld, Peter Littler, Hannah Hayhurst, Derek Manas, Ralph Jackson, John Moir, Jeremy French

**Affiliations:** 1 Department of Interventional Radiology, Freeman Hospital, Freeman Road, High Heaton, Newcastle upon Tyne, NE7 7DN UK; 2 Department of Hepatobiliary Surgery, Freeman Hospital, Newcastle upon Tyne, NE7 7DN UK

**Keywords:** Selective internal radiotherapy, SIRT, Resection after SIRT, Y90, Hepatic malignancy, Surgical resection, Radioembolization

## Abstract

**Introduction:**

Selective internal radiotherapy (SIRT) with yttrium-90 (Y-90) is an intra-arterial therapy for hepatic malignancy in patients who are unsuitable for surgical resection. This treatment is considered palliative, although some patients can demonstrate a response that is adequate to facilitate surgical resection with curative intent.

**Methods:**

All patients who underwent liver resection post SIRT were reviewed. Data gathered included patient demographics, tumor type, surgical details, and post-operative outcomes.

**Results:**

Twelve patients underwent SIRT followed by liver resection (7 males and 5 females). Pathologies were hepatocellular carcinoma (*n* = 5), metastatic colorectal cancer (*n* = 5), and neuroendocrine tumor (*n* = 2). Lesional response (size, volume, and RECIST (response evaluation criteria in solid tumors)) was calculated and where appropriate functional liver remnant (FLR) is presented. Mean FLR increase was 264cm^3^ (range − 123 to 909), and all cases demonstrated a partial response according to RECIST with a mean largest lesion volume reduction of 475cm^3^ (range 14–1632). No post-SIRT complications were noted. Hepatectomy occurred at a mean of 322 days from SIRT treatment. Ninety-day morbidity was 67% (*n* = 6), complications post-surgery were analyzed according to the Clavien-Dindo classification scale; a total of 15 events occurred in 6 patients. Ninety-day mortality of 11% (*n* = 1).

**Conclusion:**

In selected cases, liver resection is possible post SIRT. As this can represent a potentially curative option, it is important to reconsider resection in the follow-up of patients undergoing SIRT. Post-operative complications are noted following major and extended liver resection. Therefore, further studies are needed to improve patient selection.

## Introduction

Selective internal radiotherapy (SIRT) with yttrium-90 (Y90) is an intra-arterial directed therapy for hepatic malignancy. Small (20–60 μm) microspheres containing Y90, a beta emitter with a mean penetration range of 2.5 mm and a half-life of 64 h are infused into the target liver arteries in order to treat the tumors [1, 2]. As hepatic tumors are mostly supplied by the arterial system, the delivery of Y90-coated microspheres into the liver arteries has a localized brachytherapy effect, while aiming to spare normal liver parenchyma which is supplied by the portal venous system. There are two commercially available forms of Y90 microspheres; Theraspheres (BTG, London, UK) which produce glass-coated microspheres and SIR-Spheres (Sirtex, Sydney, Australia) which produce resin coated microspheres.

Most of the reported clinical evidence for SIRT is in the context of metastatic colorectal cancer (mCRC), hepatocellular carcinoma (HCC), and metastatic neuroendocrine tumors (mNET) [2]. The role of SIRT in the management of other hepatic malignancies is yet to be clearly defined [3]. For mCRC, data demonstrates SIRT can improve progression-free survival in the liver, although results on its influence on overall survival and quality of life are evolving [4–7]. In HCC, SIRT may improve survival when compared with transarterial chemoembolization (TACE) and have potentially similar overall survival compared with sorafenib, but with an improved side effect profile [8, 9]. For mNET, SIRT has been associated with a high response rate and improved survival [10].

Despite promising results, SIRT is considered a salvage therapy. A small cohort of patients can however have a response to SIRT which renders them potential candidates for curative surgical resection [11, 12]. The experience with hepatic resection post SIRT is limited with less than 100 published cases. We retrospectively reviewed our experience with this technique.

## Materials and Methods

Between 2011 and 2017, 138 Y90 deliveries were given in 107 patients. All patients underwent multi-disciplinary discussion prior to consideration of SIRT and were deemed surgically unresectable. Prior to administering SIRT, all patients received a planning angiogram and technetium ^99m^Tc macro aggregated albumin (T99mMAA) injection with subsequent gamma camera scintigraphy or CT-SPECT to detect shunting into the lungs or extrahepatic uptake. Follow-up imaging was planned at three monthly intervals post SIRT, but this did not occur in all patients. It is important to emphasize that similar to a previous study, SIRT was not intended a bridge to surgical resection at the time of treatment [12].

On subsequent follow-up imaging, a patient had response to SIRT whereby the surgical team thought surgical resection would be possible, then these cases were rediscussed at the multi-disciplinary tumor board. All patients who underwent hepatic resection post SIRT were reviewed.

Following resection, the 90-day post-operative mortality and morbidity were reviewed. In order to allow for comparison with a previous case series, morbidity was determined by Clavien-Dindo [13].

## Results

### Patients

A total of 12 (7 males, 5 females) patients underwent hepatic resection following SIRT, representing 11% of all SIRT cases. All patients had a normal serum bilirubin (5.0 to 17.0 mmol/L) and had stopped chemotherapy at least 6 weeks prior to SIRT. Outcomes for 3 patients of these patients are not included in this review as one patient lacked follow up imaging data, another had a confounding procedure (portal vein embolization), and the third had a hemi hepatectomy with palliative intent due to symptomatic carcinoid syndrome with neuroendocrine metastases. Cases performed with curative intent were reviewed. A summary of case details is provided in Tables 1, 2, 3 and 4.

**Table 1:** Demographics

Patient	Gender	Diagnosis	Tumor volume (cm^3^)	Pre-SIRT FLR	SIRT treatment	Post SIRT tumor volume	Post SIRT FLR	Change in FLR
1	F	Metastatic colorectal	35	625	SIRTEX–right lobe	21	1096	+ 275
2	F	Metastatic neuroendocrine	109	240	SIRTEX–right lobe and segment 4 (staged)	41	420	+ 180
3	M	HCC	1150	656	SIRTEX–right lobe	136	819	+ 163
4	M	HCC	998	920	SIRTEX–left lobe	21	1126	+ 206
5	F	Metastatic colorectal	33	490	SIRTEX–right lobe	9	936	+ 446
6 (Figure 1 and 2)	M	HCC	1695	560	TheraSpheres–two treatments right lobe/segment 4	63	1469	+ 909
7	M	HCC	508	1350	TheraSpheres–right lobe	306	1524	+ 174
8	M	Metastatic colorectal	22	347	SIRTEX–right lobe	5	493	+ 146
9	F	HCC (Fibrolamellar)	350	1250	TheraSpheres–bilobar split treatment	20	1127	− 123*

Volumes calculated in cm^3^

*Due to bilobar treatment, some of the SIRT dose was likely given to the FLR resulting in a reduction in overall FLR

**Fig. 1:**
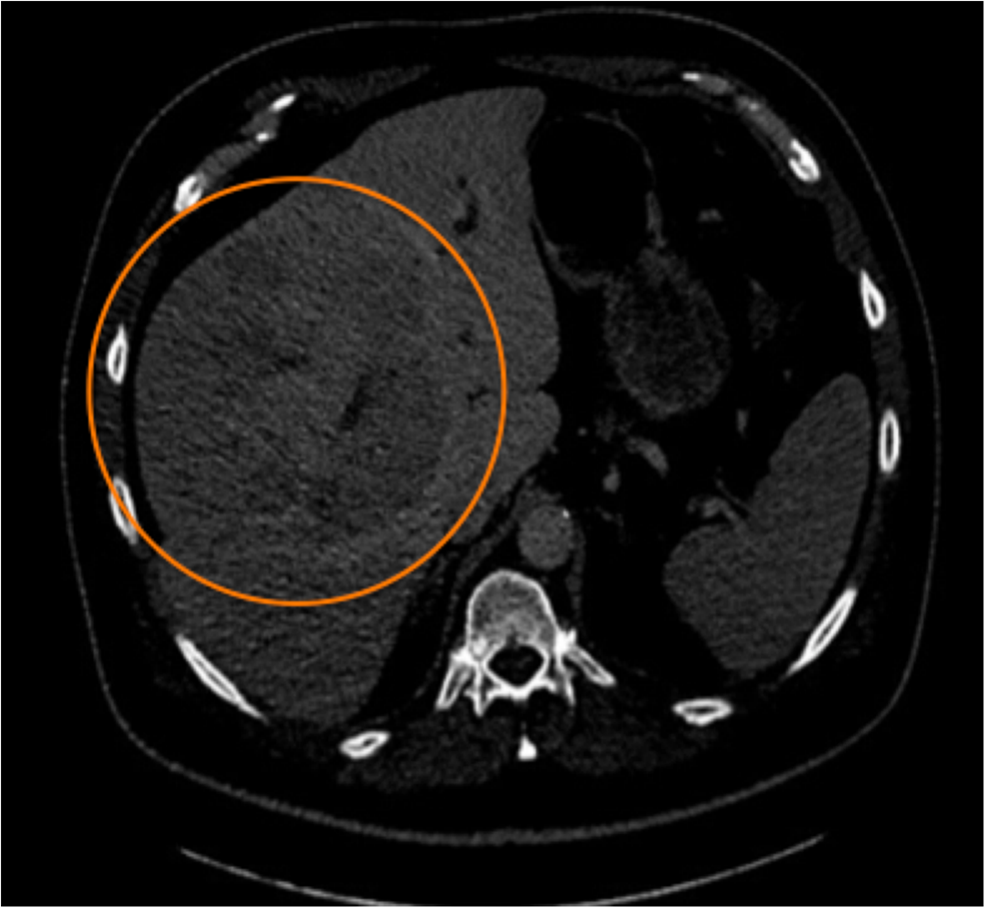
Contrast-enhanced CT of a male patient with a 14-cm HCC (orange circle) with a tumor volume of 1695 cm^3^ and inadequate FLR (560 cm^3^) for surgical resection

**Fig. 2:**
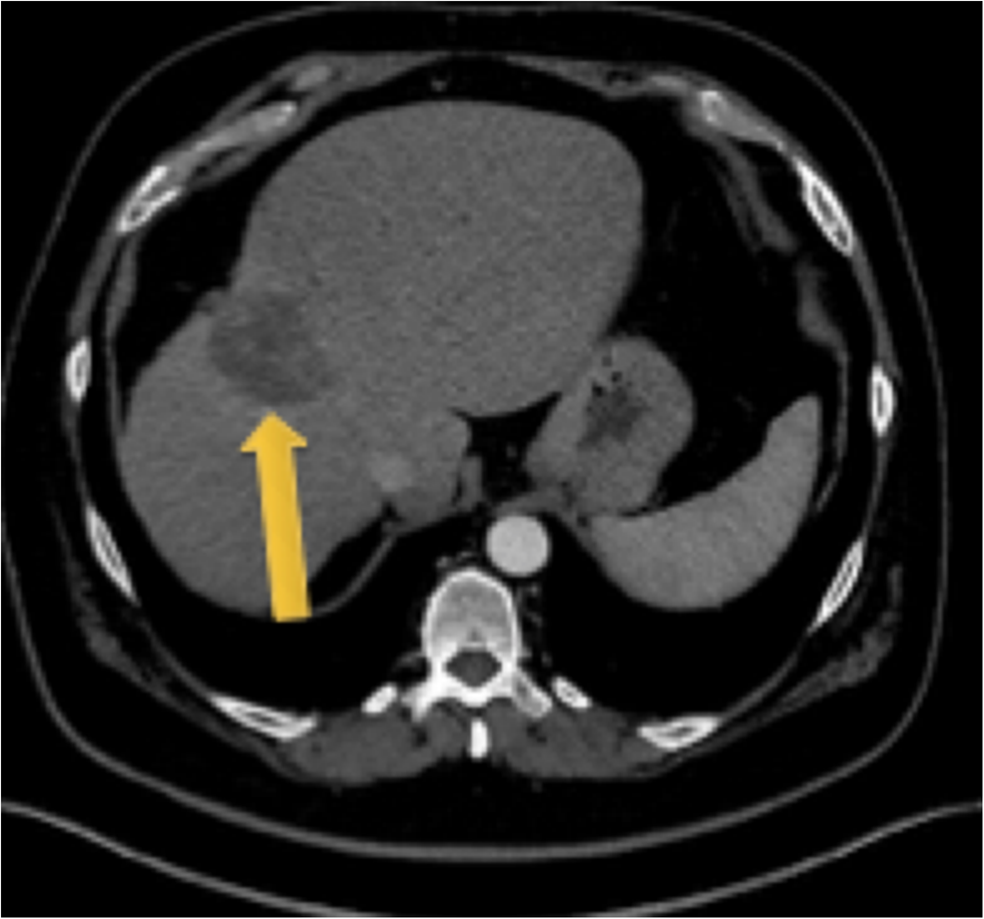
Contrast-enhanced CT following two SIRT treatments. The tumor had decreased in size to a volume of 63 cm^3^, and the FLR increased to 1469 cm^3^ rendering the patient suitable for surgical resection

**Table 2:** Surgical details

Patient	SIRT to resection (days)	Surgery	Margin
1	147	Extended right hemihepatectomy	R0
2	703	Right hemihepatectomy and metastectomy ×3 and right hemicolectomy	R1
3	195	Extended right hepatectomy	R1
4	334	Left hemi hepatectomy	R0
5	130	Extended right hepatectomy, caudae lobectomy, and IVC reconstruction	R1
6	234	Extended right hemihepatectomy and roux-en-y gastrojejunostomy (Figure 3)	R1
7	678	Right posterior sectionectomy (segment 6/7) and cholecystectomy	R0
8	218	Extended right hemihepatectomy	R1
9	256	Extended left hepatectomy with resection and reconstruction of IVC	R0

**Fig. 3:**
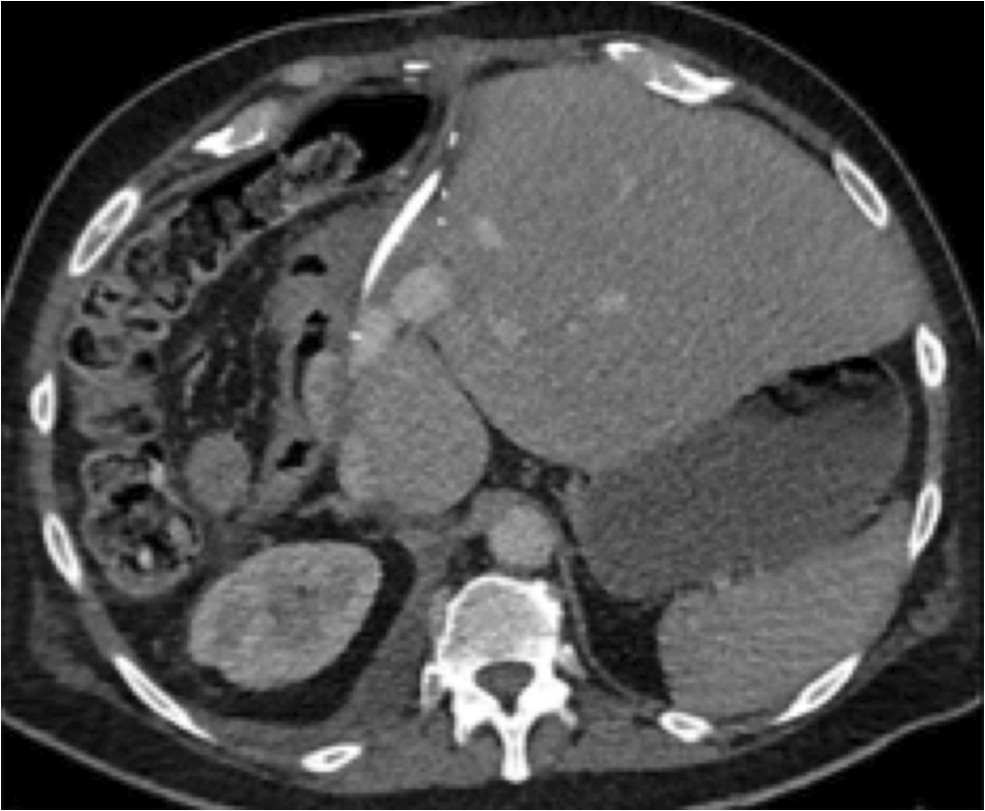
Contrast-enhanced CT following extended right hepatectomy with roux-en-y hepaticojejunostomy

**Table 3:** All Complications related to hepatic resection post SIRT

Classification (Clavien-Dindo)	Event	Number
I		
II	Pneumonia	2
	Biliary Sepsis	2
	IVC Thrombus	1
IIIa	Bile Leak	4
	Biliary sepsis requiring percutaneous decompression	1
IIIb	Bilio-pleural fistula	1
	Hepatic vein stenosis (stented)	1
IVa	Respiratory failure	1
IVb	Multi organ failure	1
V	Death	1

**Table 4:** Surgical outcomes

Patient	Length of stay	Complication	90-Day re-admission	Cause for re-admission
1	79 (died)	Hepatic vein stenosis (stented), biliary sepsis, bile leak, pneumonia, and multi-organ failure	NA	
2	20	None	No	
3	6	None	No	
4	13	None	No	
5	30	Bile leak/collection	No	
6	79	Bile leak, sepsis, respiratory failure, and bilio-pleural fistula	Yes	Rigor with displaced biliary drain
7	10	Hospital acquired pneumonia	No	
8	11	Biliary sepsis	No	
9	67	Bile leak, IVC thrombus	No	

### SIRT

Five patients underwent a single SIRT treatment while the remaining 4 patients had two treatments (this included two planned staged treatments and two repeat administrations). Eight patients had unilobar SIRT, and 1 patient received whole-liver SIRT. For the 2 patients who received a second SIRT administration, this occurred due to significant residual disease post initial SIRT, and following multi-disciplinary discussion, a second treatment was felt to be in the patients’ best interests. No SIRT-related complication was seen. All lesions demonstrated a partial response to SIRT with a mean largest lesion volume reduction of 475 cm^3^ (range 14–1632). Where functional liver remnant (FLR) was felt to be a contributory reason to being unresectable, this was also calculated pre and post SIRT with a mean increase in FLR increase of 264 cm^3^ (range − 123 to 909). One patient has a decrease in FLR (− 123 cm3) as they received a bilobar treatment where the FLR was also exposed to SIRT. All patients who were initially unresectable on multi-disciplinary review had a reduction in lesion size and/or regression from critical vascular structures and/or increase in the necessary FLR. Patients proceeded to resection only after undergoing secondary multi-disciplinary review after SIRT response.

### Surgical Outcomes

Mean time from SIRT to resection was 322 days (range 195–703). Surgical procedures were extended right hemihepatectomy (*n* = 5), right hepatectomy (*n* = 1), extended left hepatectomy (*n* = 2), and right posterior sectionectomy (*n* = 1). Two cases required resection and reconstruction of the IVC. One case also underwent a metastasectomy at resection and another a roux-en-y hepaticojejunostomy. A R0 resection was achieved in 4 patients (44%).

Median hospital stay was 16.5 days (range 6–79) with one re-admission (11%). The 90-day morbidity rate was 67% (*n* = 6) with 15 complications occurring in 6 patients, of which 10 complications were Clavien-Dindo grade 3 or above (Table 3). Ninety-day mortality was 11% (*n* = 1) where a patient died 79 days post procedure following a complicated post-operative course with chest sepsis, bile leak, hepatic vein stenosis (stented), and subsequent multi-organ failure. No cases of post-hepatectomy liver failure were seen.

At mean follow-up at 878 days [range 79-2156], three patients died of which two developed radiographic progression, 5 patients remain alive and 1 was lost to follow up.

## Discussion

With hepatic malignancy drawing its blood supply primarily from the hepatic arterial system, the delivery of Y90 microspheres into this circulation is theorized to have preferential uptake in tumor cells rather than the normal liver tissue which is supplied by the portal venous system. As a beta emitter, the Y90 loaded microspheres have an effective radiotherapy range of about 2 .5mm and induce tumor cell injury through DNA damage [2]. Due to the short effective distance of the microspheres and portal venous blood supply, normal hepatic parenchyma should be relatively spared from the Y90 microsphere’s radiotherapy effect [14].

Surgical resectability is determined by the ability to safely achieve a surgical margin (R0) while preserving a residual adequate liver volume ((FLR) future liver remnant), typically 25–30% of a healthy liver, but can increase up to 40% in patients with cirrhosis or after significant doses of chemotherapy [15]. If the FLR is the limiting factor for resection but is tumor free, portal vein embolization (PVE) can be considered to increase FLR. In the context of PVE failure, or bilobar disease, associating liver partition and portal vein ligation for staged hepatectomy (ALPPS) has been suggested as an alternative, albeit more controversial [16]. The disadvantage of both PVE and ALPPS is that while hepatic hypertrophy occurs, the initial disease process is left to progress and can even be stimulated by the hypertrophy process. Unilobar SIRT has the advantage of treating the liver tumors, which can result in volume reduction of the diseased lobe and contralateral lobe hypertrophy [17]. Furthermore, the hypertrophy occurs over an extended period of time, typically a number of months. This is reflected in the observed time from SIRT to surgery time (mean 322 days). It is thought this slower hypertrophy (compared to PVE or ALPPS) provides a better liver function per volume in the remnant liver. However, the degree of contralateral hypertrophy is typically less than can be achieved with PVE [18]. The precise mechanism for hypertrophy post SIRT is not known, but is likely multifactorial due to changes in portal venous hemodynamics and biochemical influences [19–24]. Except one The R0 resection rate of 44% in this series was despite the use of intraoperative ultrasound guiding clear resection of all of macroscopic disease. Of note, the positive margins identified after scrupulous histological examination consisted of limited microscopic infiltrative areas in areas not amenable to further surgical resection.

With its ability to both downstage disease and increase FLR, surgical resection after SIRT has in recent years been performed. Several case reports and small case series have suggested it can be a technically feasible option in carefully selected patients [25–30]. Only four larger series have also been published with a total experience of *n* = 64 [11, 12, 31, 32]. In two of these series, resection was an afterthought following SIRT [10, 11] while Justinger et al. specifically selected marginally resectable cases and performed their surgical resections at a shorter interval (approximately 2 months) following SIRT [31]. Unless specifically intended at the time of SIRT, hepatic resection following SIRT is rare, with published literature indicating between 2 and 8% of patients receiving SIRT become surgically resectable [10, 11]. More recent data from the SIRFLOX trial has suggested that up to 13% of patients with metastatic colorectal cancer can become resectable after SIRT [33]. Further analysis the SIRFLOX imaging data post SIRT has also suggested that more patients might become technically resectable than initially thought [34].

Direct comparison of our data with other series is challenging due to the heterogeneous nature of the available information. Several key themes however can be identified. Firstly, surgical resection after SIRT is associated with an increased morbidity compared to similar resections in patients who have not undergone SIRT, most commonly a bile leak. Our 90-day morbidity was in line with other studies ranging from 42 to 78% [11, 12]. It is unclear whether this high morbidity is due to pre-treatment with SIRT. Resections in cirrhotic patients with HCC can have morbidities of greater than 50% [35]. However, in our cohort, the majority of hepatic complications occurred in the mCRC group. SIRT has been proposed to theoretically increase surgical morbidity as it can induce sinusoidal obstruction. This theory is purely based on the premise that sinusoidal obstruction can also be seen after oxaliplatin-based chemotherapy which has been shown to increase operative morbidity [7, 36, 37]. SIRT can also induce adhesion formation between the liver and adjacent structures which has in one case required partial diaphragmatic resection [7, 25]. One series with HCC patients alone reported a much lower complication rate (16% Clavien-Dindo grade III or above) [32].

The impact of tumor type on surgical morbidity and the safest time for resection after SIRT are topics for further investigation. What this data and that of related studies do highlight is that SIRT should no longer be viewed only as a salvage therapy and patients’ cases should re-reviewed at multi-disciplinary tumor boards for reconsideration of resection after SIRT. For patients with inadequate FLRs, PVE remains the standard of care. However, in circumstances where a tumor risks becoming unresectable due to the tumor growth that can be induced by PVE, SIRT may be a preferential treatment [38].

## Conclusion

This study, while not changing suggested practice, contributes to the existing limited evidence indicating that hepatic resection after SIRT is technically possible albeit with a higher morbidity. It is unclear whether the higher morbidity is due to pre-treatment with SIRT. Patients demonstrating a response to SIRT should be reconsidered for resection. Multi-disciplinary tumor board decision making remains key to patient selection. More research is needed to best identify candidates who may benefit from surgical resection following SIRT.
